# Disturbance of Copper Homeostasis Is a Mechanism for Homocysteine-Induced Vascular Endothelial Cell Injury

**DOI:** 10.1371/journal.pone.0076209

**Published:** 2013-10-18

**Authors:** Daoyin Dong, Biao Wang, Wen Yin, Xueqing Ding, Jingjing Yu, Y. James Kang

**Affiliations:** 1 Regenerative Medicine Research Center, West China Hospital, Sichuan University, Chengdu, Sichuan, China; 2 Agilent Technologies (China) Co., Ltd., Beijing, China; Duke University Medical Center, United States of America

## Abstract

Elevation of serum homocysteine (Hcy) levels is a risk factor for cardiovascular diseases. Previous studies suggested that Hcy interferes with copper (Cu) metabolism in vascular endothelial cells. The present study was undertaken to test the hypothesis that Hcy-induced disturbance of Cu homeostasis leads to endothelial cell injury. Exposure of human umbilical vein endothelial cells (HUVECs) to concentrations of Hcy at 0.01, 0.1 or 1 mM resulted in a concentration-dependent decrease in cell viability and an increase in necrotic cell death. Pretreatment of the cells with a final concentration of 5 µM Cu in cultures prevented the effects of Hcy. Hcy decreased intracellular Cu concentrations. HPLC-ICP-MS analysis revealed that Hcy caused alterations in the distribution of intracellular Cu; more Cu was redistributed to low molecular weight fractions. ESI-Q-TOF detected the formation of Cu-Hcy complexes. Hcy also decreased the protein levels of Cu chaperone COX17, which was accompanied by a decrease in the activity of cytochrome c oxidase (CCO) and a collapse of mitochondrial membrane potential. These effects of Hcy were all preventable by Cu pretreatment. The study thus demonstrated that Hcy disturbs Cu homeostasis and limits the availability of Cu to critical molecules such as COX17 and CCO, leading to mitochondrial dysfunction and endothelial cell injury.

## Introduction

The link between hyperhomocysteinemia and atherosclerosis was originally proposed more than 40 years ago by McCully [1], who observed advanced arterial lesions in children with inborn errors of methionine metabolism. Since then, experimental and clinical studies have produced supporting evidence that elevated blood levels of homocysteine (Hcy) is linked to increased risk of coronary artery disease, stroke, and thromboembolism [2]–[5]. Current understanding of the association between hyperhomocysteinemia and atherosclerosis is related to a direct toxic effect of Hcy on endothelial cells, interaction between Hcy and clotting factors, and/or promotion by Hcy of oxidation of low-density lipoproteins (LDL) [5], [6].

The observation that blood copper (Cu) and Hcy were simultaneously elevated in patients with cardiovascular disease [2], [4], [7] generated interests in studying Cu and Hcy interaction and the consequence. There are several lines of evidence that indicate the importance of Cu and Hcy interaction in the increased risk for cardiovascular disease. First, it has been invariably observed that hyperhomocysteinemia is associated with high concentrations of blood Cu as well as ceruloplasmin [2], [4], [7]. Second, Cu chelator penicillamine significantly reduced the cardiovascular effects of hyperhomocysteinemia [8], [9]. Third, Cu and Hcy complexes have been identified *in vitro* and their exposure to cultured endothelial cells elicited remarkable changes in relation to atherogenic activities [10]–[13]. These observations collectively suggest that the interaction between Cu and Hcy plays an important role in vascular endothelial injury.

There is virtually no free Cu in mammalian cells [14]. The intracellular trafficking of Cu is tightly regulated by Cu chaperones [15], [16]. The Cu chaperones directly or indirectly acquire Cu from Cu transporters such as Ctr-1 and Ctr-2 on the mammalian cell membrane. Among these Cu chaperones is COX17, which delivers Cu to COX-11, Sco1, or Sco-2, through which cytochrome c oxidase (CCO) receives Cu for the enzyme assembly and function. Therefore, disturbance of intracellular Cu homeostasis would result in changes in Cu transport to the critical molecules such as COX17 and CCO, leading to mitochondrial dysfunction, and eventually cell injury. The present study was thus undertaken to test the hypothesis that disturbance in Cu homeostasis is a mechanism for Hcy-induced endothelial cell injury, focusing on the COX17-CCO-mitochondrial function pathway.

## Materials and Methods

### Cell culture and treatment

Human umbilical vein endothelial cells (HUVECs) obtained from American Tissue Culture Collection (ATCC) were maintained at 37°C in L-DMEM (GIBCO, USA) media supplemented with 10% fetal bovine serum (FBS, Hyclone) and 1% penicillin/streptomycin (GIBCO, USA) in 5% CO_2_ incubator. Stock cultures were maintained at 80% confluence and passaged by 0.25% Trypsin (GIBCO, USA) and 1% EDTA in Ca^2+^- and Mg^2+^-free phosphate-buffered saline (PBS). Experimental cells were subcultured in 25 cm^2^ flasks at 2×10^5^ cells/flask overnight. Cells were treated for 24 hrs with 0.01, 0.1, or 1 mM D, L-homocysteine (Hcy) (Sigma, USA) or/and 5 µM CuSO_4_ in FBS-free L-DMEM when the cell density reached to about 30% confluence. Hcy and CuSO_4_ were dissolved in deionized water and sterile filtered before they were added to the cultures. Cells grown on a 25/75 cm^2^ flasks were scraped and washed twice with PBS, and pelleted in nondenaturing lysis buffer (pH 7.6, 20 mM Tris-HCl buffer, 150 mM NaCl, 20 mM KCl, 1.5 mM MgCl_2_, 0.2% NP-40, protease inhibitors:10 µg/µL leupeptin, and 5 µg/µL aprotinin, 1 mM PMSF). The cell pellet was cold-treated on ice for 30 min and vortexed three times. All the steps were in N_2_ gas protection and kept in ice to prevent oxidation. Cell lysates were used for Western Blot, HPCL-ICP-MS.

### MTT assay

Cell viability was assessed by measuring the mitochondrion-dependent reduction of MTT ((3, 4, 5-dimethylthiazol-2-yl)-2, 5-diphenyltetrazolium bromide) to formazan. Briefly, MTT was dissolved in PBS in 5 mg/ml and cells were subcultured for 24 hrs in 96-well-plate containing L-DMEM supplemented with 10% FBS. Before adding MTT, media were replaced with flesh FBS-free L-DMEM and MTT was added directly and incubated for 4 hrs. At the end, the media were removed and 200 µl dimethylsulfoxime (DMSO) was added to dissolve purple formazan. The absorbance at 570 was monitored using a μQuant (BioTek, USA) spectrophotometer equipped with KCjunior software.

### Lactate dehydrogenase (LDH) release assay

LDH released from cells was determined as an index of cell necrosis, following a method described previously [17]. Briefly, substrate was prepared as follows: dissolve 1.21 g of Tris (Bio-Rad, USA), 0.28 g of sodium lactate (Sigma, USA) in 25 ml of distilled water, adjust pH to 8.2 (25°C) with hydrochloric acid (HCl), and make the final volume of 50 ml with distilled water. The final sodium lactate concentration was 50 mM. The preparation of colorimetric reagent was as follows: dissolve 4 mg of 2-p-iodophenyl-3-pnitrophenyl-5-phenyl tetrazolium chloride (INT, Sigma, USA), 10 mg of nicotinamide adenine dinucleotide (NAD, Sigma, USA), and 1 mg of phenazine methosulfate (PMS, Sigma, USA) in 2 ml distilled water. The measurement procedure included: adding 170 µl substrate, 10 µl sample, and 30 µl colorimetric reagent into 96-well plate. Absorbance (OD) at 503 nm at time 0 and 25 min was recorded using the above-mentioned spectrophotometer. The extent of LDH release from cells was calculated as follows equation.
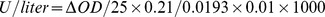



### Determination of intracellular Hcy concentrations

Hcy was measured by using high pressure liquid chromatography (HPLC) as described earlier [18]. HPLC analyses were performed using Waters Millenium system (Waters 600) with a Waters 474 fluorescent detector and a Supelcosil LC 18 DB analytical column (250 mm×4.6 mm, 3 µM particle size) along with a pre-column. The temperature inside the column was maintained at 25°C during elution. The sample preparation was as follows: into an aliquant of 90 µl cell lysate, added 10 µl reducing agent and 10% tris (2-carboxyethyl) phosphine hydrochloride (TCEP, Sigma) at 4°C and maintained for 30 min. The precipitation of proteins was achieved by addition of 100 µl methanol and subsequent centrifugation for 10 min at 2000× g. The supernatant of 100 µl was labeled with a fluorescent marker, 10 µl of 7-fuorobenzo-2-oxa-1,3-diazole-4-sulphonic acid (SBD-F, Sigma), 10 mg/ml solution in 0.125 M borate buffer, pH 9.5. The mixture was incubated at 60°C for 60 min and subjected to HPLC separation and analysis. The HPLC was operated as follows: mobile phase was 93% 0.05 M KH_2_PO_4_, adjusted pH to 2.1 with ortho-phosphoric acid and 7% acetonitrile. Flow-rate was 1.0 ml/min and total elution time was 20 min. Fluorescent was monitored at an emission wavelength of 515 nm and excitation wavelength of 385 nm. Calibration was based on external standard using Hcy diluted in distilled water and peak height was used for calculation of concentrations.

### Determination of intracellular Cu concentrations

Intracellular Cu concentrations were determined by a graphite furnace atomic absorption spectrophotometer (AAS). Briefly, cells were collected by cell scraper, washed tree times with PBS, and centrifuged at 500 g for 15 min. The precipitation was freeze-dried and dissolved in 50 µl concentrated HNO_3_ for 3 days. At the time of sample analysis, 0.5 ml deionized water was added before AAS measurement.

### Cu distribution assay by HPLC-ICP-MS

An aliquot of 20 µl cell lysate sample was applied to a gel filtration column (TSK-GEL G2000SW_XL_ 300×7.5 mm; TOSOH, Japan). The column was eluted with 50 mM Tris–HCl, pH 7.4, at a flow rate of 1 mL/min. A mixture of protein standards for SEC column calibration was obtained from Bio-Rad Laboratories (Hercules, CA, USA), which contained thyroglobulin (670 kDa), γ-globulin (158 kDa), ovalbumin (44 kDa), myoglobin (17 kDa), and vitamin B12 (1.35 kDa). The elute was introduced directly into the nebulizer tube of an Agilent 7700 ICP-MS (Agilent Technologies, USA) and Cu was monitored at m/z 63. The parameters for the operation of ICP-MS were as follows: plasma RF power, 1500 W; plasma gas flow, 15.0 L/min; auxiliary gas flow, 1.15 L/min; nebulizer gas flow, 1.05 L/min; dwell time, 100 ms; and point per peak, 1.

### Identification of Cu-Hcy complexes by ESI-Q-TOF

An equal volume (100 µL) of each 100 µM Hcy and 100 µM CuSO_4_ was mixed at room temperature for 2 hrs. The samples were analyzed by ESI-Q-TOF. The ion source was operated in a positive ion mode. Mass spectrometer calibration was performed using an ESI-L low concentration Tuning Mix (Agilent technologies, USA). The optimum setting was: ion source voltage 3.5 kV, capillary temperature 350°C, source heater temperature 120°C, nitrogen sheath gas flow 20 (arbitrary unit) and auxiliary gas flow 5 (arbitrary unit), nebuilizer 30 psig, fragmentor 120 V, skimmer 65 V, OCT 1RF Vpp 250 V. Mass spectra were collected using data dependent acquisition of one MS full scan (100–1000 m/z).

### Mitochondrial membrane potential assay

An reagent, 5,5′,6,6′- tetrachloro-1,1′,3,3′ tetraethylbenzimidazoly-carbocyanine iodide (JC-1), was used to assess mitochondrial potential changes using confocal microscope. Briefly, cells in 24-well plates at a density of 1×10^5^ cells/ml cultured at 37°C overnight were treated with Hcy and/or Cu for 24 hrs before addition of 1 µg/ml JC-1 in culture media for 30 min. The cells were examined by confocal microscope (Nikon, Japan). Changes in the fluorescence from red to green indicate the changes in mitochondrial membrane potential; the green/red ratio was calculated from the changes in the intensity of red and green fluorescence assessed using a computer program.

### Determination of cytochrome c oxidase (CCO) activity

Mitochondria were isolated from collected cells using a mitochondrial isolation kit (Pierce, #89801) following the instruction provided. Bio-Rad protein assay was used to measure mitochondrial protein concentrations. An aliquot of 0.25 mg mitochondrial protein was prepared in 0.2 ml of isosmotic medium containing 10 mM KH_2_PO_4_, pH 6.5, 50 mM KCl, 0.25 M sucrose, 1 mg/ml BSA, and 2.5 mM n-dodecylmaltoside. CCO activity was determined by adding 0.2 mM ferrocytochrome c. The enzyme activity was calculated from the rate of decrease in absorbance of reduced cytochrome c at 550 mM (ε = 19.1 mmol-1 cm-1). Because accurate estimation of CCO activity could be compromised by variations in mitochondrial yield and integrity, the CCO activity in the present study was normalized using an invariant marker of mitochondrial enzyme activity, citrate synthase. Citrate synthase was assayed by following the reduction of 0.1 mM 5,5′- dithio-bis (2-nitrobenzoic acid) in the presence of 0.25 mg mitochondrial protein, 0.25 mM acetyl-CoA, and 0.5 mM oxalacetid acid in a medium with 40 mM PB, pH 8.0, 2 mM EDTA, 1 mg/ml BSA, and 0.1% Triton X-10. The change in the absorbance at 412 nm (ε = 13.6 mmol-1 cm-1) was monitored for 30 min (reflecting the formation of thionitrobenzoate).

### Western blotting analysis of protein concentrations

The protein contents of COX17 were determined by Western blot. Cells scraped in PBS were washed 3 times and lysated using 1% SDS solution. Protein samples were mixed with 5× loading buffer, boiled for 10 min at 100°C and cooled. Equal amounts of protein (50–100 µg) from each sample were separated by 12% SDS-PAGE. Proteins were then electrophoretically transferred to a polyvinylidene fluoride membrane (Bio-Rad, USA). Transferred proteins were blocked with 5% non-fat dry milk in Tris-HCl buffer solution containing Tris-HCl (50 mM), NaCl (150 mM), and Tween-20 (0.1%) (TBS-T) for 1 hr at room temperature. The blots were then incubated with respective primary antibodies (anti-COX17 and anti-Atox1, Abcam, USA; anti-CCS, Santa Cruz, USA) in blocking solution according to the vender's recommendations. After incubation, the blots were washed with TBS-T six times for 5 min each. The blots were incubated for 2 hrs with appropriate secondary antibody. After washing six times (5 min each), target proteins were visualized using chemiluminescence (Bio-rad, USA) and analyzed by densitometry using a Quantity One Software.

### Statistics

**Figure 1:**
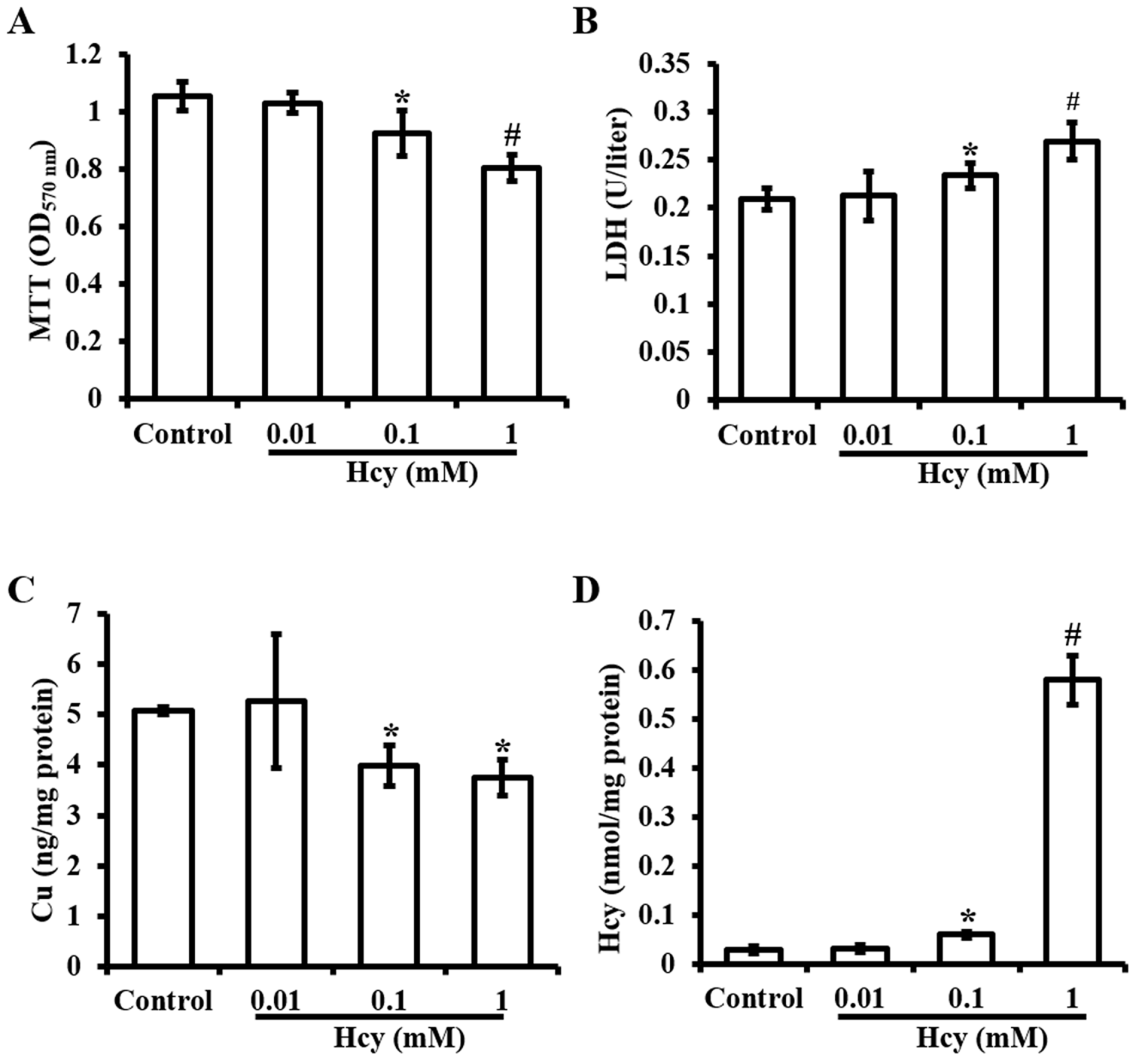
Homocysteine (Hcy) induced cell viability changes in and LDH release from human umbilical vein endothelial cells (HUVECs). **A.** MTT assay of cell viability changes as a function of Hcy concentrations. **B.** Measurement of LDH in media (LDH release) after treatment with concentrations of Hcy. **C.** Intracellular concentrations of Cu after 24 hrs exposure to concentrations of Hcy in cultures. **D.** Intracellular concentrations of Hcy after 24 hrs exposure to concentrations of Hcy in cultures. Each data point was obtained from three independent experiments and each experiment contains triplicate samples for each treatment. Values are means ±S.E.M. * or # significantly different from control group and from each other (p<0.05).

**Figure 2:**
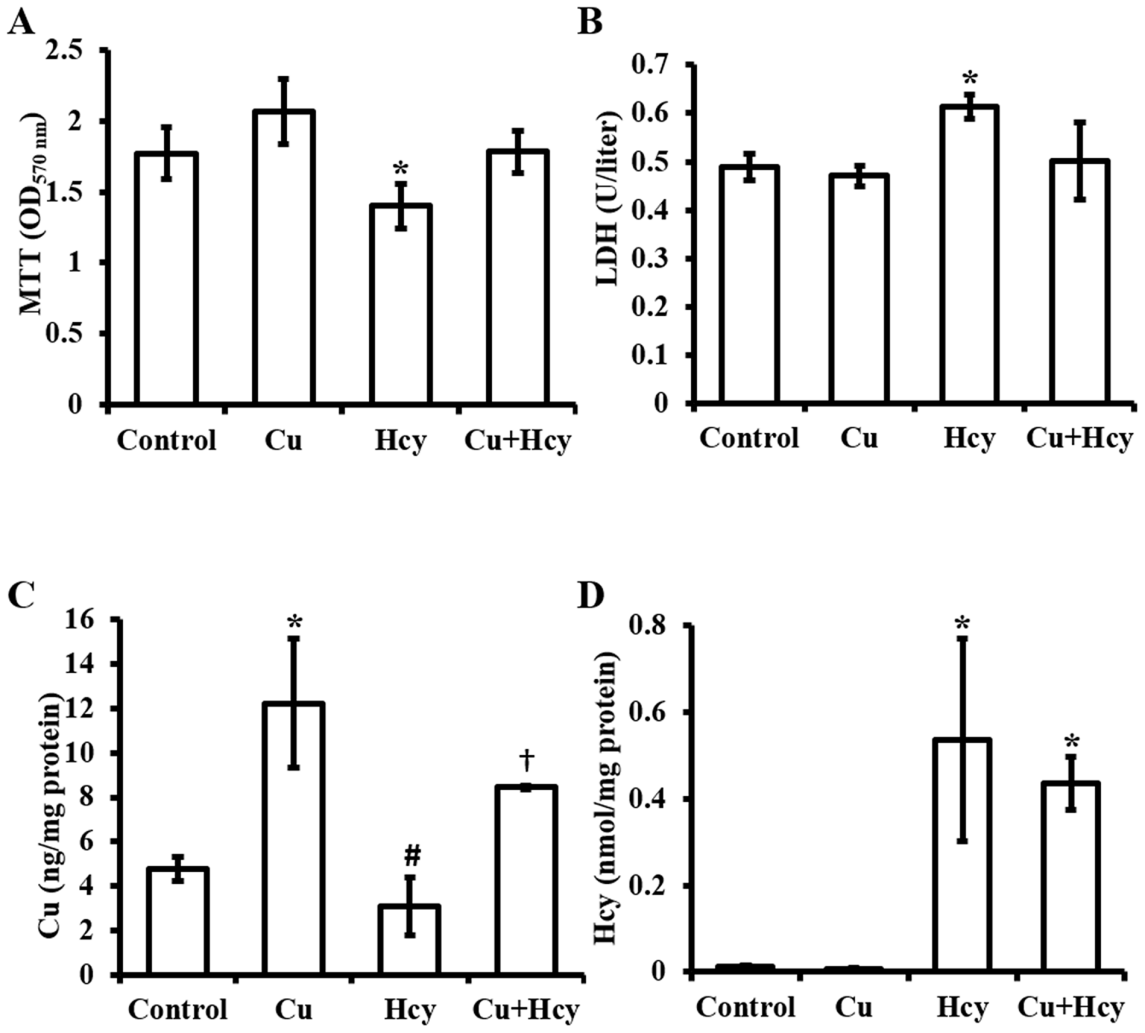
Effects of Cu (5 µM) pretreatment on Hcy-induced decrease in cell viability and LDH release. **A.** MTT assay of cell viability changes. **B.** Measurement of LDH in media (LDH release). **C.** Intracellular concentrations of Cu. **D.** Intracellular concentrations of Hcy. Each data point was obtained from three independent experiments and each experiment contains triplicate samples for each treatment. Values are means ±S.E.M. *, # or † significantly different from control group and from each other (p<0.05).

**Figure 3:**
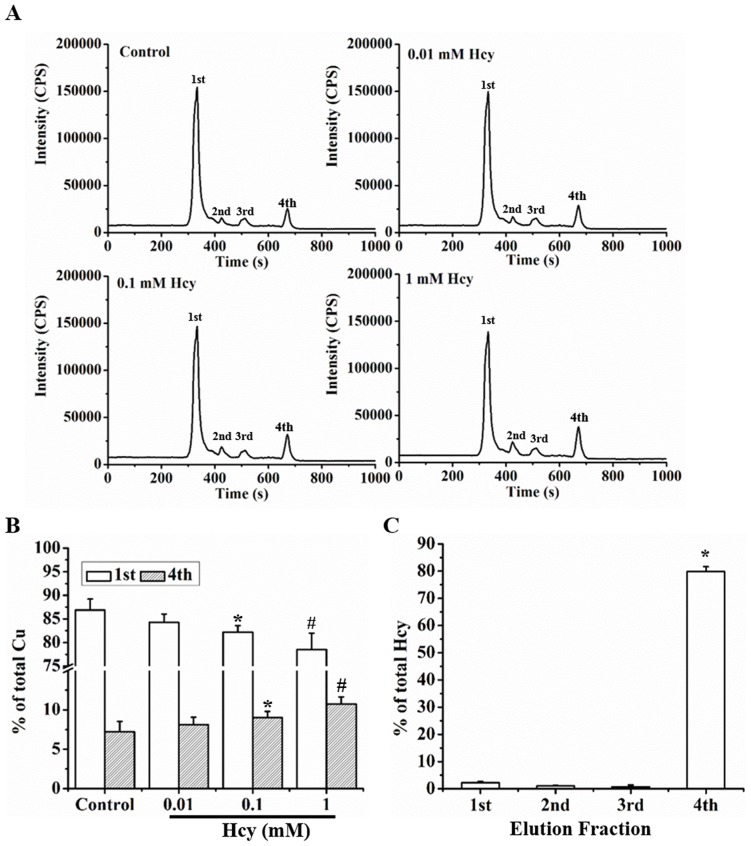
Hcy induced redistribution of cellular Cu. **A.** Measurement of cellular Cu distribution after treatment with concentrations of Hcy by HPLC-ICP-MS. **B.** Hcy-induced redistribution of Cu between high (1^st^) and low (4^th^) molecular weight fractions as evaluated by changes in percentage of Cu in each fraction. **C.** Distribution of Hcy in each fraction. Each data point was obtained from three independent experiments and each experiment contains triplicate samples for each treatment. Values are means ±S.E.M. * or # significantly different from control group and from each other for different fractions (p<0.05).

**Figure 5:**
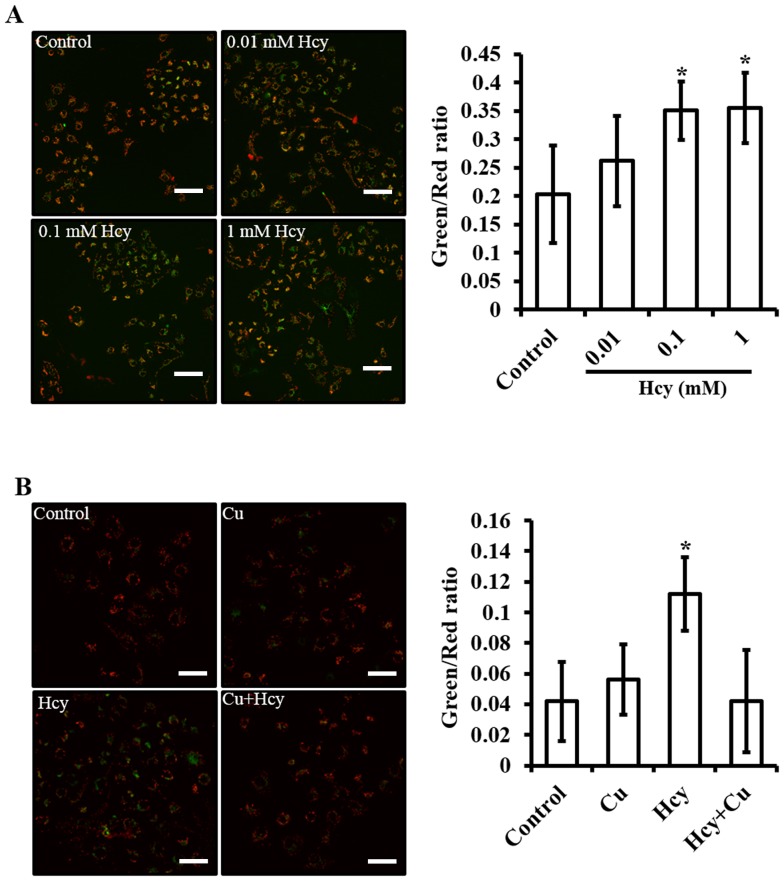
Effect of Hcy on mitochondrial membrane potential (ΔΨm) in HUVECs. **A.** JC-1 assay of mitochondrial membrane potential changes as a function of Hcy concentrations. **B.** The effect of Cu pretreatment on Hcy-induced mitochondrial membrane potential changes. Bar: 100 µm. Each data point was obtained from three independent experiments and each experiment contains triplicate samples for each treatment. Values are means ±S.E.M. * significantly different from control group (p<0.05).

**Figure 6:**
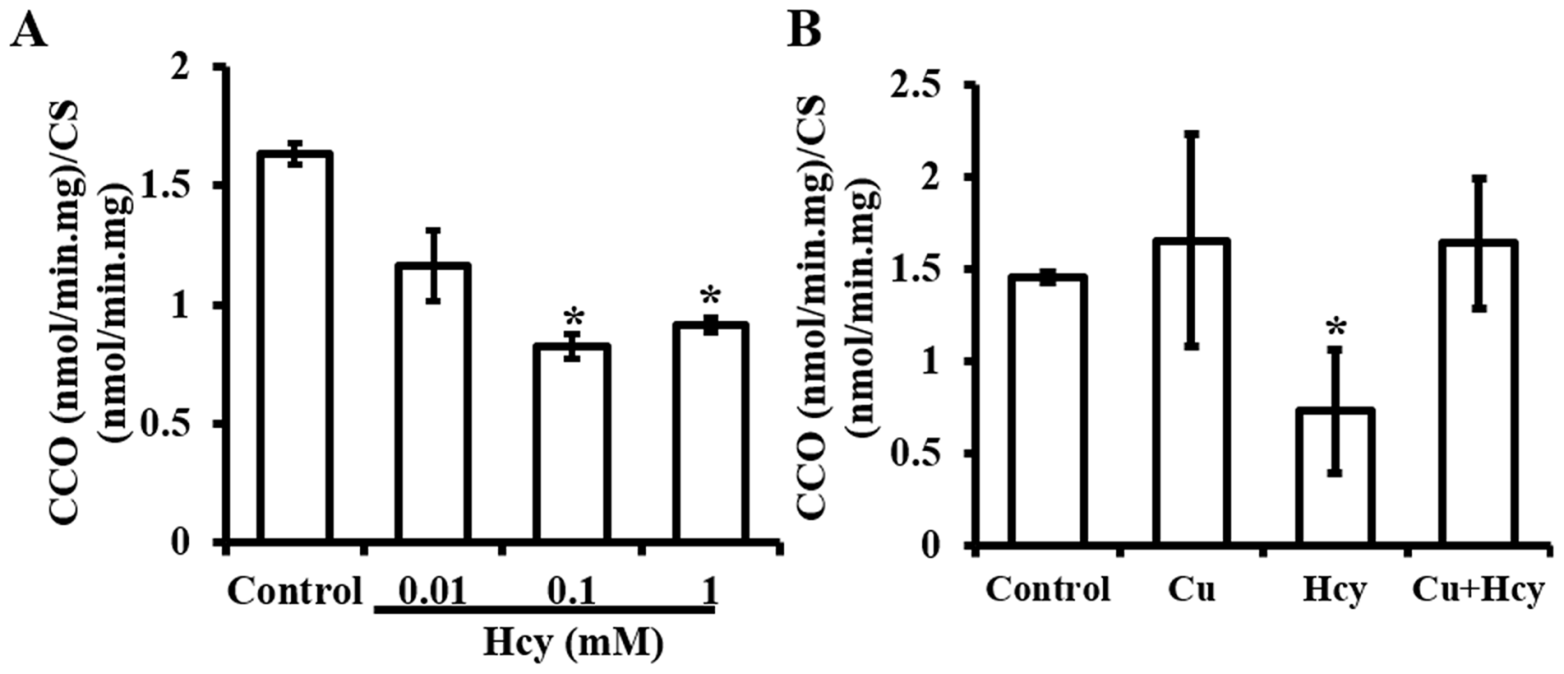
Enzymatic assay for changes in cytochrome c oxidase (CCO) activity. **A.** Changes in the CCO activity as a function of Hcy concentrations. **B.** The effect of Cu pretreatment on Hcy-induced changes in the CCO activity. The treatment protocol and labels are the same as described for Figure 2. Each data point was obtained from three independent experiments and each experiment contains triplicate samples for each treatment. Values are means ±S.E.M. * significantly different from control group (p<0.05).

**Figure 7:**
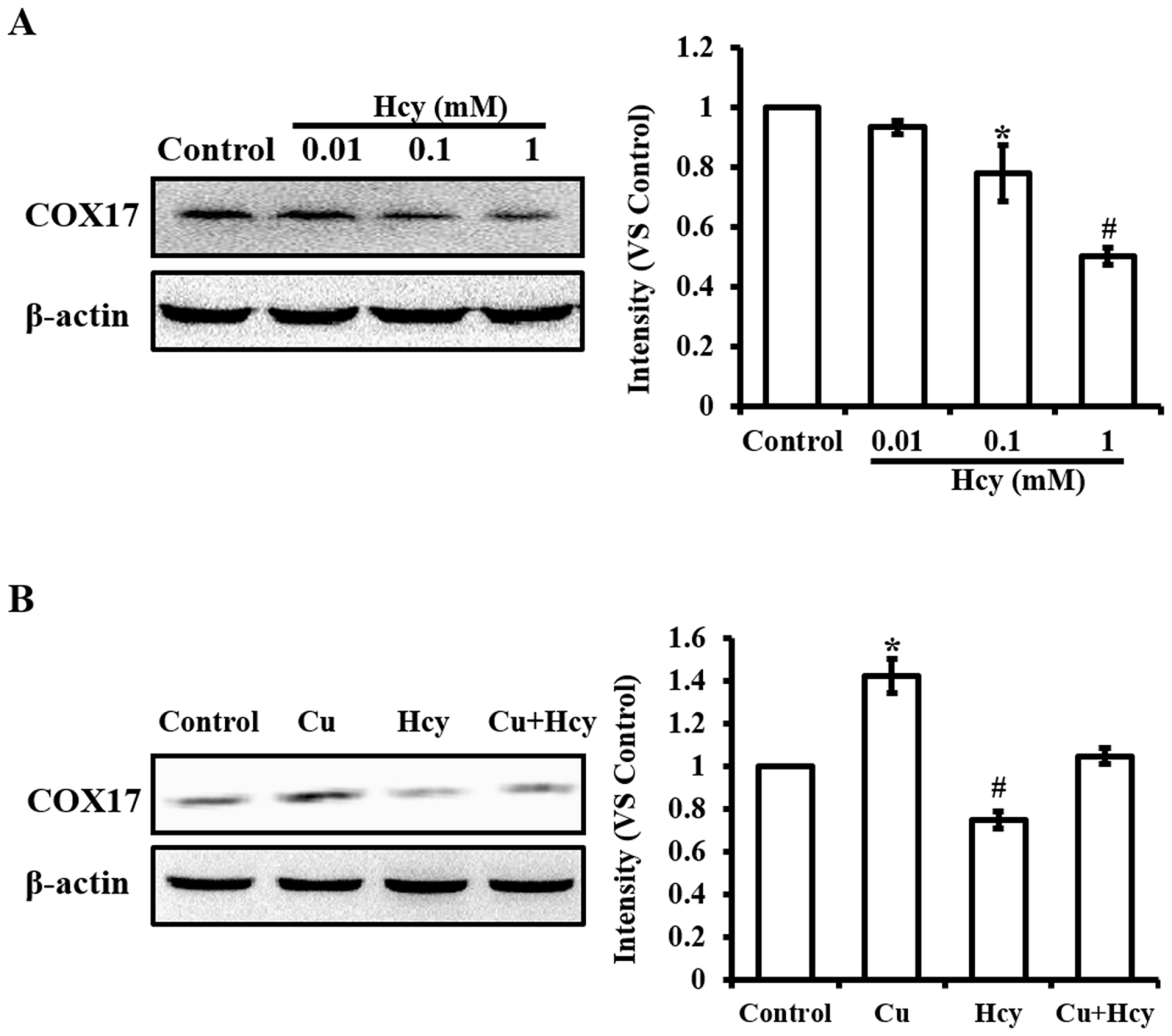
Western blot analysis of Hcy-induced changes in COX17 protein level. **A.** Changes in the COX17 protein level as a function of Hcy concentrations. **B.** The effect of Cu pretreatment on Hcy-induced changes in the COX17 protein level. The treatment protocol and labels are the same as described for Figure 2. Semiquantitative analyses based on the density changes of each protein on the blot were obtained from 6 independent blots. Values are means ±S.E.M. * or # significantly different from control group and from each other (p<0.05).

Data were obtained from three separate experiments and presented as mean values ± S.E.M. One-way ANOVA was used for initial analysis and Dunnett's test was employed for comparison among multiple groups for the data presented in Fig. 1, 3, 5A, 6A, and 7A. A 2×2 factorial design was applied to the data presented in Fig. 2, 5B, 6B, and 7B, and the significance of differences between main effects and interaction was determined by *F*-test. Differences were considered significant at *P*<0.05.

## Results

### Effects of Hcy on cell viability, cell necrosis, and intracellular Cu concentrations

Exposure of HUVEC cells to 0.01, 0.1, or 1.0 mM Hcy caused a concentration-dependent decrease in cell viability, as determined by a MTT assay (Fig. 1A). Further analysis revealed that Hcy also caused a concentration-dependent increase in cell necrosis determined by LDH release assay (Fig. 1B). In addition, intracellular Cu concentrations were decreased in Hcy-treated cells (Fig. 1C) along with a significant increase in intracellular Hcy concentrations (Fig. 1D). In the cells pretreated with 5 µM Cu, the effects of Hcy (0.1 mM) on cell viability (Fig. 2A), LDH release (Fig. 2B), and the loss of intracellular Cu contents (Fig. 2C) were blocked. But this Cu pretreatment did not affect cellular accumulation of Hcy (Fig. 2D).

### The effect of Hcy on Cu partitions to different molecules in the cell

Cell lysates were subjected to HPLC-ICP-MS analysis as described in the Methods section. There were four major Cu-partition fractions identified, and a shift caused by Hcy in Cu distribution to these fractions was observed. A reduction of Cu distribution to high molecular weight fractions occurred after the cells were treated with Hcy at concentrations above 0.1 mM, which was accompanied by an increase in Cu distribution to the low molecular weight fraction, as shown in Fig. 3A and 3B. In the low molecular weight fraction, Hcy concentrations were significantly increased in the cells treated with Hcy at concentrations above 0.1 mM (Fig. 3C).

### Formation of Cu-Hcy complexes *in vitro*

**Figure 4:**
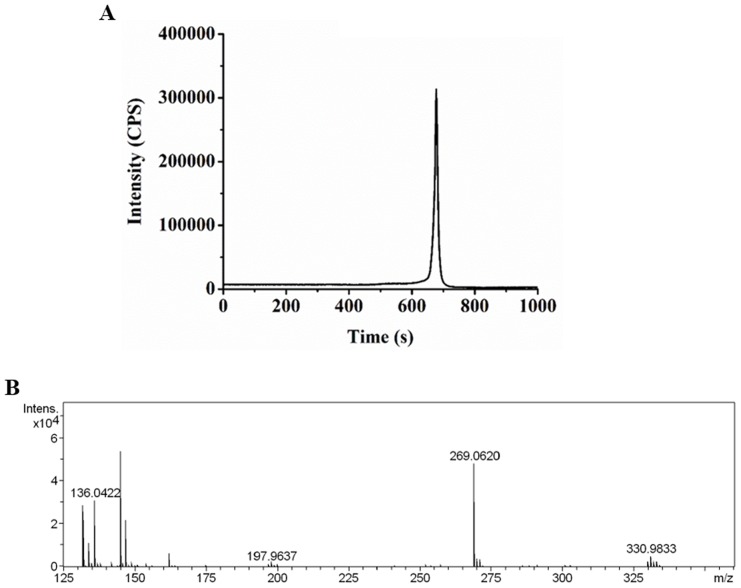
Mass spectrometric analysis of complex formation between Cu and Hcy. **A.** Chromatogram of Cu and Hcy mixture. **B.** ESI mass spectrum of Cu and Hcy mixture. The histogram identifies homocysteine (136.0422), Cu-homocysteine (197.9637) homocystine (269.0620), and Cu-homocystine (330.9833), as shown in the schematic structures (Table 1).

We made an attempt to measure intracellular Cu-Hcy complexes in order to further demonstrate the relationship between changes in Cu homeostasis and Hcy exposure. However, it was difficult at present due to the limitation of currently available methods and the unknown kinetics of Cu-Hcy complexes *in vivo*. On the other hand, we detected the interaction between Cu and Hcy *in vitro*. The formation of Cu-Hcy complexes *in vitro* was analyzed by ESI-Q-TOF. Cu-homocysteine and Cu-homocystine complexes were identified as shown in Fig. 4 and Table 1. The intense signal in the spectrum at m/z 330.9833 was corresponding to the Cu-homocystine complex, and at m/z 197.9637 corresponding to Cu-homocysteine.

**Table 1:** Chemical formula of homocysteine, Cu-homocysteine complex, homocystine and Cu-homocystine complex.

No.	Chemical name	Chemical formula	Estimated value	Theoretical value	Error (ppm)
1	homocysteine	C_4_H_9_O_2_NS	136.0422	136.0427	3.675
2	Cu-homocysteine complex	C_4_H_7_O_2_NSCu	197.9637	197.9645	4.041
3	homocystine	C_8_H_16_O_4_N_2_S_2_	269.0620	269.0624	1.487
4	Cu-homocystine complex	C_8_H_14_O_4_N_2_S_2_Cu	330.9833	330.9847	4.230

### The effect of Hcy on mitochondrial membrane potential (ΔΨm)

We measured the changes in mitochondrial membrane potential (ΔΨm) using confocal microscope assisted by JC-1 fluorescence. As shown in Fig. 5, the control cells showed a polarized ΔΨm with more red than green JC-1 fluorescence. The treatment with 0.1 mM Hcy caused a remarkable shift from polarized to depolarized ΔΨm, as indicated by the increase in green fluorescence over red. Pretreatment with Cu did not cause noticeable changes in mitochondrial membrane potential, but eliminated the shift induced by Hcy from red to green.

### Effects of Hcy on CCO activities and protein contents of COX17

The results presented in Fig. 6A showed that treatment with 0.1 mM Hcy significantly decreased CCO activities in the cells. Pretreatment with 5 µM Cu did not cause changes in basic activities of CCO, but attenuated the inhibitory effect of Hcy on CCO activities (Fig. 6B). COX17 is a Cu chaperone that transfers Cu to CCO via COX-11, Sco1 or Sco2; therefore, changes in COX17 affect the downstream events in Cu transfer. Western blot analysis was used to determine changes in COX17 protein after treatment with Hcy in cells. The analysis of proteins isolated from the cells treated with concentrations of Hcy identified that COX17 was significantly decreased as a function of Hcy concentrations in cultures (Fig. 7A). To define whether the changes in COX17 caused by Hcy exposure can be blocked by Cu pretreatment, the proteins isolated from cells pretreated with Cu prior to Hcy exposure in comparison with the cells treated with Hcy or Cu only were analyzed by Western blot. The results in Fig. 7B show that Cu treatment alone increased the level of COX17, and Cu pretreatment blocked the inhibitory effect of Hcy on COX17 protein contents.

## Discussion

The data obtained from this study showed that the exposure of HUVECs to Hcy at clinically relevant concentrations caused a concentration-dependent endothelial cell injury. Treatment with Hcy resulted in an accumulation of intracellular Hcy, along with a decrease in Cu concentrations and a redistribution of Cu in the cell. The increase of Hcy concentrations in low molecular weight components was associated with the increased Cu partition, indicating the interaction between Cu and Hcy, which was further evidenced by the formation of Cu-Hcy complexes *in vitro*. The consequence of the alteration by Hcy of Cu intracellular homeostasis included depressed activities of Cu-dependent mitochondrial enzyme CCO, decreased protein levels of Cu chaperone for CCO, COX17, and the collapse of mitochondrial membrane potential. Collectively, these results demonstrate that disturbance of Cu homeostasis leads to the limitation of Cu to critical molecules such as COX17 and CCO involved in mitochondrial integrity and function, making a significant contribution to Hcy-induced endothelial cell injury.

The clinical definition of hyperhomocysteinemia is based on the measurement of serum Hcy levels from the blood drawn after a 12-hour fast: levels between 5 and 15 µmol/L are considered normal, between 16 and 30 are classified as moderate, 31 and 100 as intermediate, and greater than 100 as severe elevation [15]. Therefore, the level of 0.1 mM Hcy in cultures would be equivalent to an intermediate elevation of Hcy in the blood. The result presented here showed that at the clinically relevant level of 0.1 mM Hcy in cultures, the HUVEC cells underwent detrimental alterations in cell viability and necrosis. These changes in the endothelial cells would reflect the effect of hyperhomocyteinemia *in vivo* on vascular endothelium in clinical settings, in which high levels of Hcy in the blood caused a direct toxic effect on endothelial cells [5]. Although this toxic effect is often ascribed to oxidative injury induced by Hcy through its intracellular metabolism, the observation presented in the present study indicates an alternative mechanism by which Hcy causes metabolic disorder of Cu in the endothelial cells.

The formation of Cu-Hcy complexes had been known [10]–[13], which was confirmed in the present study. But the effect of Cu-Hcy complexes on endothelial cells has not been understood. There were at least two adverse events caused by Hcy in Cu homeostasis observed in the present study. One was the decrease in intracellular Cu concentrations and the other was the alteration of Cu distribution; namely Cu redistribution from high molecular weight components to low molecular weight components. While it is difficult to speculate at present the relationship between Cu loss and the formation of Cu-Hcy complexes, the redistribution of Cu would be related to the formation of Cu-Hcy complexes. In the low molecular weight fraction in which Cu concentrations were increased, Hcy levels were significantly increased. This would indicate that the formation of Cu-Hcy complexes was at least partially responsible for Cu redistribution in the endothelial cells.

This redistribution of Cu to low molecular weight components limited the availability of Cu to high molecular weight components, leading to dysfunction of critical Cu-dependent proteins. As evidenced by the observation in this study, the activity of mitochondrial Cu-dependent enzyme CCO were significantly depressed. Since this enzyme is critically involved in the last step of electron transfer of mitochondrial respiratory chain reaction, it is predictable that depression of CCO activities leads to damages to mitochondrial integrity and function. This was demonstrated by the result showing that CCO depression was accompanied by a collapse of mitochondrial membrane potential in the Hcy-treated cells. This observation is in an agreement with an early report [13] that the binding of Cu and Hcy is responsible for Hcy-induced CCO depression and apoptosis in neural cells.

COX17 is a critical Cu chaperone to transfer Cu, via COX-11, Sco1 or Sco2, to CCO. Interestingly, a decrease in COX17 protein contents in Hcy-treated cells was observed in the present study. This decrease would be related to the limitation of Cu availability to this Cu chaperone. Mammalian COX17 binds co-operatively 4 Cu^+^ ions and it can exist in the form of Cu_4_COX17, partially oxidized (two disulfide bridges), or fully oxidized (three disulfide bridges) [19]. Partially oxidized COX17 can bind one Cu^+^ or Zn^2+^ ion and fully oxidized does not bind metals [19]. Metals can be released from COX17, e.g., Cu transfer from COX17 to partner proteins, by non-oxidative and oxidative mechanisms. It has been demonstrated that metal binding proteins, such as Cu and Zn binding protein metallothionein, are quickly degraded if they are not bound to metals or in the oxidized form [20]. Hcy-induced limitation of Cu availability would accelerate the process of COX17 degradation, leading to reduced cellular levels of this protein. The Hcy concentration-dependent reduction of COX17 protein levels observed in the present study would support this speculation, although it needs further investigation in future studies.

In contrast to the present observation, an early study has shown that dietary Cu deficiency did not change the protein levels of Cu transporter-1 and COX17, but increased Cu chaperone for SOD1 and Sco1, and decreased COX-I and COX-IV protein levels in rat cardiac tissue [21]. This discrepancy may result from tissue-specificity or variations between *in vivo* and *in vitro* responses. However, the reduction of COX17 protein levels would result from the limitation of available Cu rather than specific effect of Hcy, as indicated by the prevention of this effect by pretreatment with excess Cu.

The depressed COX17-CCO-mitochondrial function consequence would result from Cu limitation due to either Cu loss from the cell or redistribution to low molecular weight components. This was proven by the fact that pretreatment with excess Cu attenuated all of the adverse effects of Hcy treatment. It should be noticed that the Cu concentration (5 µM) used in the present study was physiologically relevant. Therefore, the availability of labile Cu is more important than the total amount of Cu in the cell. Although the formation of Cu-Hcy complexes would directly limit the availability of Cu, it is unknown whether other metabolic alterations induced by Hcy also restrict Cu intracellular trafficking. Furthermore, the specificity and mechanism of the sensitivity of COX17 and CCO to Hcy-induced limitation of Cu availability are important topics for future studies.

In summary, the present study provided evidence that demonstrates that disturbance of Cu homeostasis by high but clinically relevant levels of Hcy is a mechanism for the adverse effect of Hcy on endothelial cells. This effect was mediated by the limitation of the availability of Cu to critical molecules such as COX17 and CCO involved in mitochondrial integrity and function, leading to mitochondrial damage and cell injury.
